# Detecting forest response to droughts with global observations of vegetation water content

**DOI:** 10.1111/gcb.15872

**Published:** 2021-09-25

**Authors:** Alexandra G. Konings, Sassan S. Saatchi, Christian Frankenberg, Michael Keller, Victor Leshyk, William R. L. Anderegg, Vincent Humphrey, Ashley M. Matheny, Anna Trugman, Lawren Sack, Elizabeth Agee, Mallory L. Barnes, Oliver Binks, Kerry Cawse‐Nicholson, Bradley O. Christoffersen, Dara Entekhabi, Pierre Gentine, Nataniel M. Holtzman, Gabriel G. Katul, Yanlan Liu, Marcos Longo, Jordi Martinez‐Vilalta, Nate McDowell, Patrick Meir, Maurizio Mencuccini, Assaad Mrad, Kimberly A. Novick, Rafael S. Oliveira, Paul Siqueira, Susan C. Steele‐Dunne, David R. Thompson, Yujie Wang, Richard Wehr, Jeffrey D. Wood, Xiangtao Xu, Pieter A. Zuidema

**Affiliations:** ^1^ Stanford University Stanford CA USA; ^2^ Jet Propulsion Laboratory California Institute of Technology Pasadena CA USA; ^3^ California Institute of Technology Pasadena CA USA; ^4^ United States Forest Service Washington DC USA; ^5^ Northern Arizona University Flagstaff AZ USA; ^6^ University of Utah Salt Lake City UT USA; ^7^ University of Texas ‐ Austin Austin TX USA; ^8^ University of California ‐ Santa Barbara Santa Barbara CA USA; ^9^ University of California ‐ Los Angeles Los Angeles CA USA; ^10^ Oak Ridge National Laboratory Oak Ridge TN USA; ^11^ Indiana University‐Bloomington Bloomington IN USA; ^12^ The Australian National University Canberra ACT Australia; ^13^ University of Texas – Rio Grande Edinburg TX USA; ^14^ Massachusetts Institute of Technology Cambridge MA USA; ^15^ Columbia University New York NY USA; ^16^ Duke University Durham NC USA; ^17^ The Ohio State University Columbus OH USA; ^18^ Centre de Recerca Ecològica i Aplicacions Forestals (CREAF) Barcelona Spain; ^19^ Universitat Autònoma de Barcelona Barcelona Spain; ^20^ Pacific Northwest National Laboratory Richland WA USA; ^21^ Washington State University Pullman WA USA; ^22^ University of Edinburgh Edinburgh UK; ^23^ Institució Catalana de Recerca i Estudis Avançats (ICREA) Barcelona Spain; ^24^ University of California ‐ Irvine Irvine CA USA; ^25^ University of Campinas Campinas Brazil; ^26^ University of Massachusetts ‐ Amherst Amherst MA USA; ^27^ Delft University of Technology Delft The Netherlands; ^28^ University of Arizona Tucson AZ USA; ^29^ University of Missouri Columbia MO USA; ^30^ Cornell University Ithaca NY USA; ^31^ Wageningen University Wageningen The Netherlands

**Keywords:** drought response, drought‐induced tree mortality, microwave remote sensing, pressure–volume, vegetation optical depth, vegetation water content, water potential

## Abstract

Droughts in a warming climate have become more common and more extreme, making understanding forest responses to water stress increasingly pressing. Analysis of water stress in trees has long focused on water potential in xylem and leaves, which influences stomatal closure and water flow through the soil‐plant‐atmosphere continuum. At the same time, changes of vegetation water content (VWC) are linked to a range of tree responses, including fluxes of water and carbon, mortality, flammability, and more. Unlike water potential, which requires demanding *in situ* measurements, VWC can be retrieved from remote sensing measurements, particularly at microwave frequencies using radar and radiometry. Here, we highlight key frontiers through which VWC has the potential to significantly increase our understanding of forest responses to water stress. To validate remote sensing observations of VWC at landscape scale and to better relate them to data assimilation model parameters, we introduce an ecosystem‐scale analog of the pressure–volume curve, the non‐linear relationship between average leaf or branch water potential and water content commonly used in plant hydraulics. The sources of variability in these ecosystem‐scale pressure‐volume curves and their relationship to forest response to water stress are discussed. We further show to what extent diel, seasonal, and decadal dynamics of VWC reflect variations in different processes relating the tree response to water stress. VWC can also be used for inferring belowground conditions—which are difficult to impossible to observe directly. Lastly, we discuss how a dedicated geostationary spaceborne observational system for VWC, when combined with existing datasets, can capture diel and seasonal water dynamics to advance the science and applications of global forest vulnerability to future droughts.

## Graphical abstract

Changes of vegetation water content (VWC) are linked to a range of tree responses to drought, including fluxes of water and carbon, mortality, flammability, and more, and can be retrieved from microwave remote sensing measurements. We highlight key frontiers through which remotely sensed VWC has the potential to significantly increase our understanding of forest responses to water stress. We argue that separate consideration of diel, seasonal, and decadal timescales can facilitate interpretation of VWC measurements for different process studies, and that VWC observations can be useful for constraining belowground water uptake. To link remotely sensed VWC estimates to plant hydraulic models, the utility and interpretation of ecosystem‐scale pressure‐volume curves are discussed.

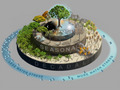

## 1 INTRODUCTION

As the climate warms, droughts are getting hotter, more extreme, and more frequent (Dai, 2013; Touma et al., 2015; Trenberth et al., 2014). Forests respond to drought by reducing photosynthesis and transpiration (Liu et al., 2020; Short Gianotti et al., 2019; Trugman et al., 2018) and exhibiting increased mortality (Adams et al., 2017; Choat et al., 2018). Their response to drought is mediated by the flow and distribution of water in the soil‐plant‐atmosphere continuum (Tyree & Sperry, 1989), but is also affected by stand‐scale processes such as belowground redistribution of water, composition, competition, and demography. These processes have been studied at fine scales (Brodribb et al., 2020; Sperry & Love, 2015), but aggregated processes controlling ecosystem response to droughts remain poorly understood (Allen et al., 2015; Gazol et al., 2018; Levine et al., 2016). Thus, we are challenged to understand forest resilience in response to the major disturbances brought by climate change (Anderegg et al., 2013; Brodribb et al., 2020), including effects on forest ecosystem services such as carbon sequestration (Anderegg et al., 2020) and water cycling (Mastrotheodoros et al., 2020; Tague et al., 2019).

Understanding the aggregated effects of drought on forests is difficult, particularly due to extensive heterogeneity in plant traits (Anderegg, 2015; Skelton et al., 2015), edaphic conditions, and topography (Goulden & Bales, 2019). Remote sensing tools naturally provide aggregate observations across ecosystems at local to global scales. Commonly used retrievals of surface temperature, leaf area, or solar‐induced fluorescence provide information on the consequences of forest drought responses (Deshayes et al., 2006; West et al., 2019), but they do not directly capture the drought stress affecting the trees themselves. These measurements also do not provide information on belowground processes affecting water redistribution and root access, a critical influence on forest drought response (Agee et al., 2021; Hagedorn et al., 2016; Phillips et al., 2016).

Microwave remote sensing of vegetation water content has become an increasingly popular alternative for studying forest responses to drought (Anderegg et al., 2018; Rao et al., 2019; Saatchi et al., 2013; Schroeder et al., 2016). For decades, numerous studies of forest–water interactions have focused on plant water potential. However, recently there has been a renewed interest in the role of vegetation water content (VWC) in influencing water and carbon fluxes, tree mortality, and fire risk (Martinez‐Vilalta et al., 2019; Matheny et al., 2015; Nolan et al., 2020). Microwave remote sensing‐based estimates of VWC (and proxies of VWC) may thus transform studies of forest responses to drought stress. However, because VWC is a relatively new remote sensing product, its optimal interpretation and use, as well as its relation to water stress, has not been comprehensively explored.

Here, we review key frontiers through which remotely sensed observations of VWC can be used to significantly increase our understanding of forest ecosystem response to droughts. We pose questions and raise challenges to maximize the utility of microwave remote sensing for studies of forest drought responses. While we focus on forests, many of the ideas in this paper also apply to other natural biomes, and particularly to detecting water stress in croplands (Steele‐Dunne et al., 2017; Togliatti et al., 2019). In Section 2, we introduce and compare plant water potential and plant water content at the tree scale, including a description of measurement challenges for each. We argue that observations of ecosystem‐scale VWC would inform a number of ecological applications and overcome several existing *in situ* measurement challenges. The ecosystem‐scale VWC measurements that are feasible from remote sensing are then introduced in Section 3. This section aims to provide some background on the theoretical basis of microwave remote sensing of VWC, in order to clarify how it informs measurement characteristics such as the canopy depth represented. This section also includes a description of challenges for improved estimation of VWC, including how additional *in situ* observations may help improve estimation accuracy. Having introduced both the in situ and remotely sensed conceptualizations of VWC, Sections 3, 4, 5, 6 of the paper then discuss the ways that remotely sensed VWC can be used to study forest response to droughts. Section 4 discusses how the current generation of remotely sensed VWC estimates can be interpreted at different timescales to provide information about different drought response processes (e.g., disturbance dynamics, canopy dehydration, etc) and the ways VWC datasets can be combined with other observations and plant hydraulic models. Because using VWC to constrain plant hydraulic models requires determining how VWC changes translate to the water potential variables that plant hydraulic models simulate, Section 5 discusses whether VWC observations can be linked to the concept of an ecosystem‐scale water potential, how such a variable could be interpreted and used, and what controls these linkages. Section 6 then discusses a specific component of forest drought response for which VWC observations can be particularly useful: determining belowground processes based on model inversion and the analysis of phase dynamics. Lastly, Section 7 considers the mismatch between available remote sensing data for VWC and the dataset properties that this paper identifies as particularly useful for studies of forest drought response (high spatial resolution, capturing diel variations). It then presents an alternative concept for a new satellite mission to address these data gaps.

## 2 FOREST DROUGHT RESPONSES—PHYSIOLOGY, POTENTIAL, AND WATER CONTENT

### 2.1 Water potential gradients influence forest drought response

The movement of water through trees (and other vascular plants) is dictated by a continuum of water potential gradients from the soil through the various plant components to the atmosphere. Variations in the magnitude of the gradient dictate how trees respond to water stress. Under dry conditions, water loss through stomata causes leaf water potential declines. As a result, stomata close, reducing the water loss but also decreasing photosynthesis. Under a drying atmosphere and/or soil, xylem water potential also decreases. At very large negative pressure (tension) in the xylem, embolisms can form, blocking water flow within the xylem vessels (Tyree & Sperry, 1989). Such embolisms reduce conductance to water transport, largely reducing the capacity to transport water from the soil to leaves. High conductance losses can potentially lead to tree mortality (Brodribb, 2009). Long‐term reductions in photosynthesis due to water stress can also make trees more vulnerable to death from some combination of biotic attack and physiological failure (McDowell, 2011; Trugman, Detto, et al., 2018; Wu et al., 2018).

### 2.2 VWC as an indicator of plant water status

**FIGURE 1:**
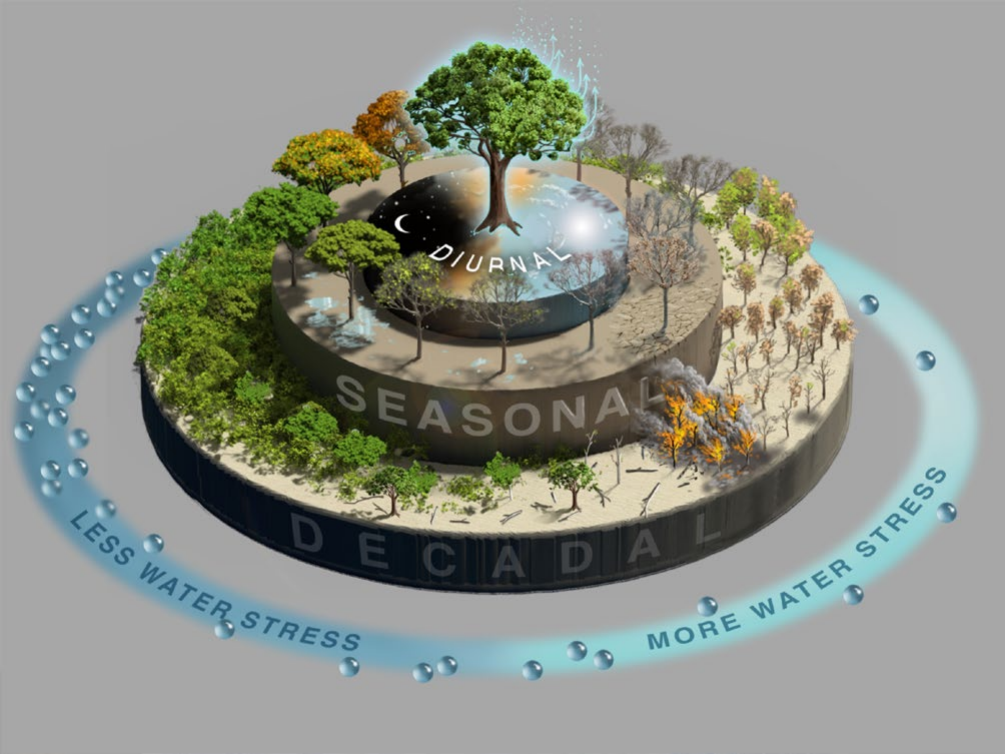
Changes in water content drive forest changes at diurnal (inner ring), seasonal (middle ring), and decadal (outer ring) timescales. Across decadal‐scale responses, declines in VWC can lead to mortality and/or fire. VWC will also increase in concert with successional dynamics. Across dry and wet seasons, forest VWC evolves through both phenology and de‐/rehydration. Lastly, VWC has a strong diurnal cycle driven by the diurnal cycle of ET

While studies of plant water relations predominantly focus on quantifying water potential variations across the soil‐plant‐atmosphere continuum, there is significant evidence that water content itself can also be an informative metric of water status. For example, plant water storage forms a significant fraction of transpiration (Goldstein et al., 1998; Matheny, Fiorella, et al., 2017; Phillips et al., 2003). In addition, the relative water content (defined by normalizing VWC by its maximum value) also provides a threshold‐based predictor for wilting and mortality under drought (Bartlett et al., 2012; Rao et al., 2019; Sapes et al., 2019). Unlike for leaf water potential, the threshold of relative water content at the wilting point was found to be relatively conservative across species (Bartlett et al., 2012). Lastly, VWC is directly related to live fuel moisture content (Konings et al., 2019; Rao et al., 2020), which is defined as the VWC per unit dry biomass and is a widely used indicator of fire risk. LFMC shows threshold‐like impacts on fire ignition probability (Chuvieco et al., 2011; Dimitrakopoulos & Papaioannou, 2001) and fire size (Argañaraz et al., 2018; Dennison & Moritz, 2009). Overall, improved quantification of VWC will likely contribute to better assessment of forest drought responses including transpiration, mortality, and wildfire risk (Figure 1). Given the large variations of VWC and relative water content within different tree components and across species, this quantification must take into account variations in VWC across vertical and horizontal scales.

### 2.3 Measurement challenges for plant water status

For decades, manual plant water potential observations (e.g., using a pressure chamber, Scholander et al., 1965) have played a central role in our understanding and quantification of tree water status, especially at the site level (Cavender‐Bares & Bazzaz, 2000; Tardieu & Simonneau, 1998; Tyree & Sperry, 1988). However, they are destructive measurements that are particularly challenging in tall forests where access to canopies leaves is limited. Consequently, these data are typically collected at weekly to monthly temporal resolutions, which may be sufficient to resolve dynamics linked to soil water drying, but are too coarse to capture variability in plant water status at diurnal timescales. Continuous, automated measurement of stem and leaf water potential is possible for some species with psychrometry (e.g., Guo et al., 2020), but these instruments are expensive, require substantial maintenance, and their use is not yet widespread. Moreover, unlike observations of water fluxes possible using micrometeorological and sap flux approaches, time series of plant water potential have yet to be collected and standardized in databases and networks, hindering synthesis of information across sites. Finally, even within a site, leaf and stem water potential measurements are also generally limited to individual trees, and scaling to the entire stand can be challenging, particularly in ecosystems with multiple species across multiple edaphic conditions. This difficulty in scaling hinders efforts to harmonize species‐specific observations to those from eddy covariance flux towers (which typically have footprints on the orders of 10^3^–10^7^ m^2^, Chu et al., 2021). These measurement difficulties also determine the scarcity of information about water potential–water content relationships (usually determined using pressure–volume curves or P‐V curves) across species and tree components. Because P‐V curves are most often determined destructively, information on P‐V curves is relatively abundant only for leaves, while very little is known about the equivalent properties of bark tissues and roots.

Direct measurements of vegetation water content can be less labor intensive and more cost effective, facilitating increased spatial and temporal observation. For wood water content, micron‐scale dendrometers can be automated and used to infer water content after detrending (Peters et al., 2021; Pfautsch et al., 2015). Reflectometry (TDR and FDR) and capacitance‐style sensors can provide automated measurements of dielectric permittivity, which can be directly converted to water content (Holbrook & Sinclair, 1992; Matheny et al., 2015; Wullschleger et al., 1996). However, these sensors are sensitive to differences in wood density, and should be calibrated for use in different species (Matheny et al., 2017). Unlike for woody tree components, there is no commonly used direct, non‐destructive measurement technique to determine leaf water content. In situ spectroscopy is sensitive to leaf water content (Browne et al., 2020; de Jong et al., 2014), but also requires species‐specific calibration. At larger scales, VWC from microwave remote sensing could be used instead of ground measurements.

## 3 MICROWAVE REMOTE SENSING OF VWC

**FIGURE 2:**
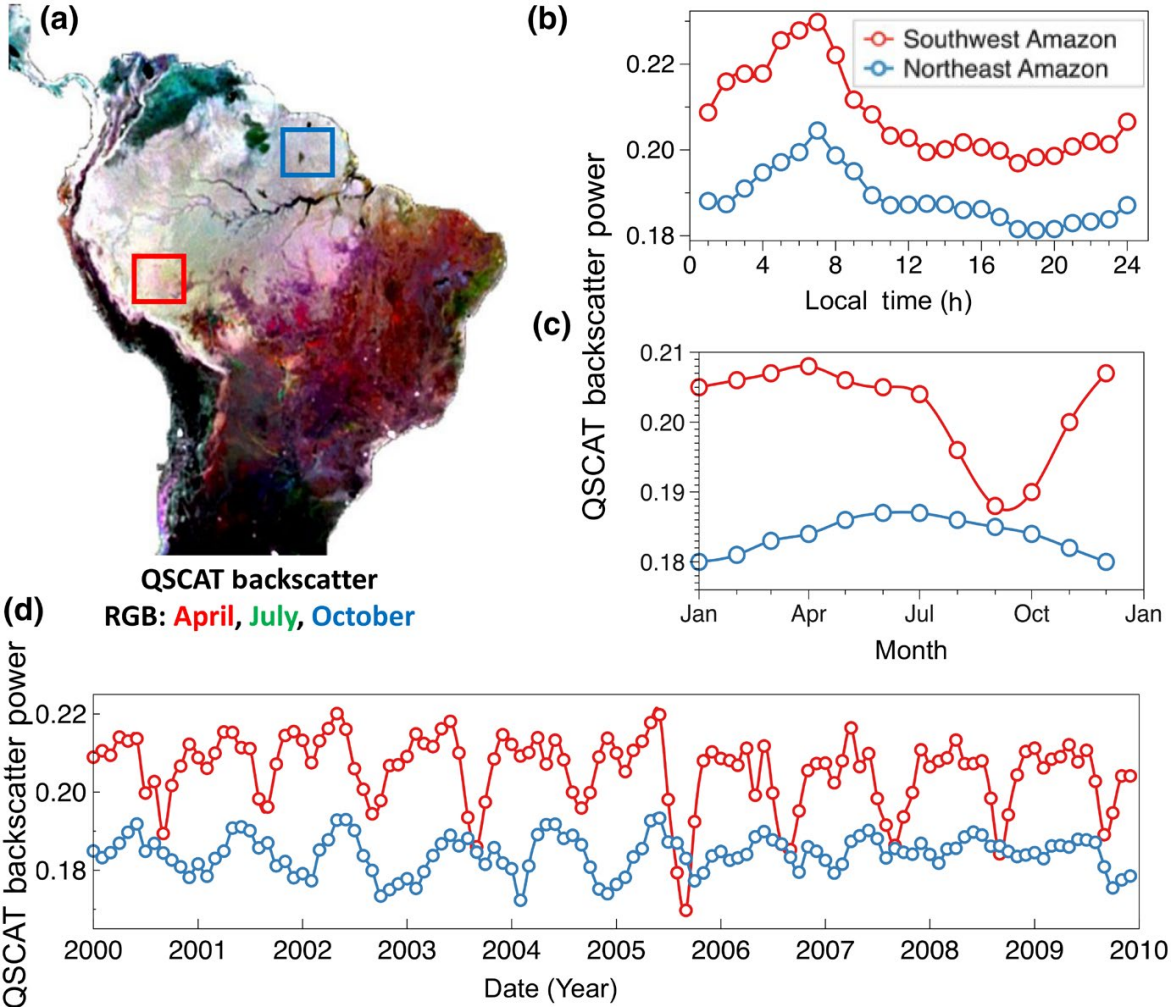
Variations of radar backscatter measurements across the Amazon Basin. Radar backscatter coefficients at Ku‐band are used as a proxy for changes of canopy water content showing: (a) spatial variations as an RGB color composite of QuikSCAT (QSCAT) radar backscatter in the months of April, July, and October capturing regional and seasonal changes, (b) seasonal cycle of QSCAT backscatter averaged across two regions in southwest and northeast of the Amazon, (c) diurnal cycle of the same regions detected by the RapidSCAT satellite observations onboard International Space Station from 2014 to 2016, and (d) time series of QSCAT backscatter capturing seasonal and interannual variations including extreme droughts of 2005 in the southwest of the Amazon

Several remote sensing techniques allow monitoring VWC or proxy measures of VWC with different levels of precision. These measurements cover a wide range of the electromagnetic spectrum, ranging from optical spectral imaging (Asner et al., 2016; Ustin et al., 2012) to thermal infrared imaging (Jones et al., 2009), to active (radar) and passive (radiometer) microwave sensing (Konings et al., 2019; Vermunt et al., 2020). Microwave frequencies are arguably the most useful for systematic measurement of VWC because of their all‐time observational capabilities during day and night and irrespective of cloud cover, and the ability to penetrate beyond the top few millimeters of the forest canopy. This avoids systematic biases that would occur if only cloud‐free periods can be measured. Observations illustrating the sensitivity of microwave remote sensing observations of VWC at different timescales are shown in Figure 2. However, no current spaceborne system is dedicated to systematically observing VWC and its changes due to water stress. We discuss prospects for a new spaceborne system, with the aim to provide estimates of VWC at sub‐daily temporal resolutions to resolve the dynamic physiological response of vegetation to water stress, in Section 7.

### 3.1 Theoretical basis

**FIGURE 3:**
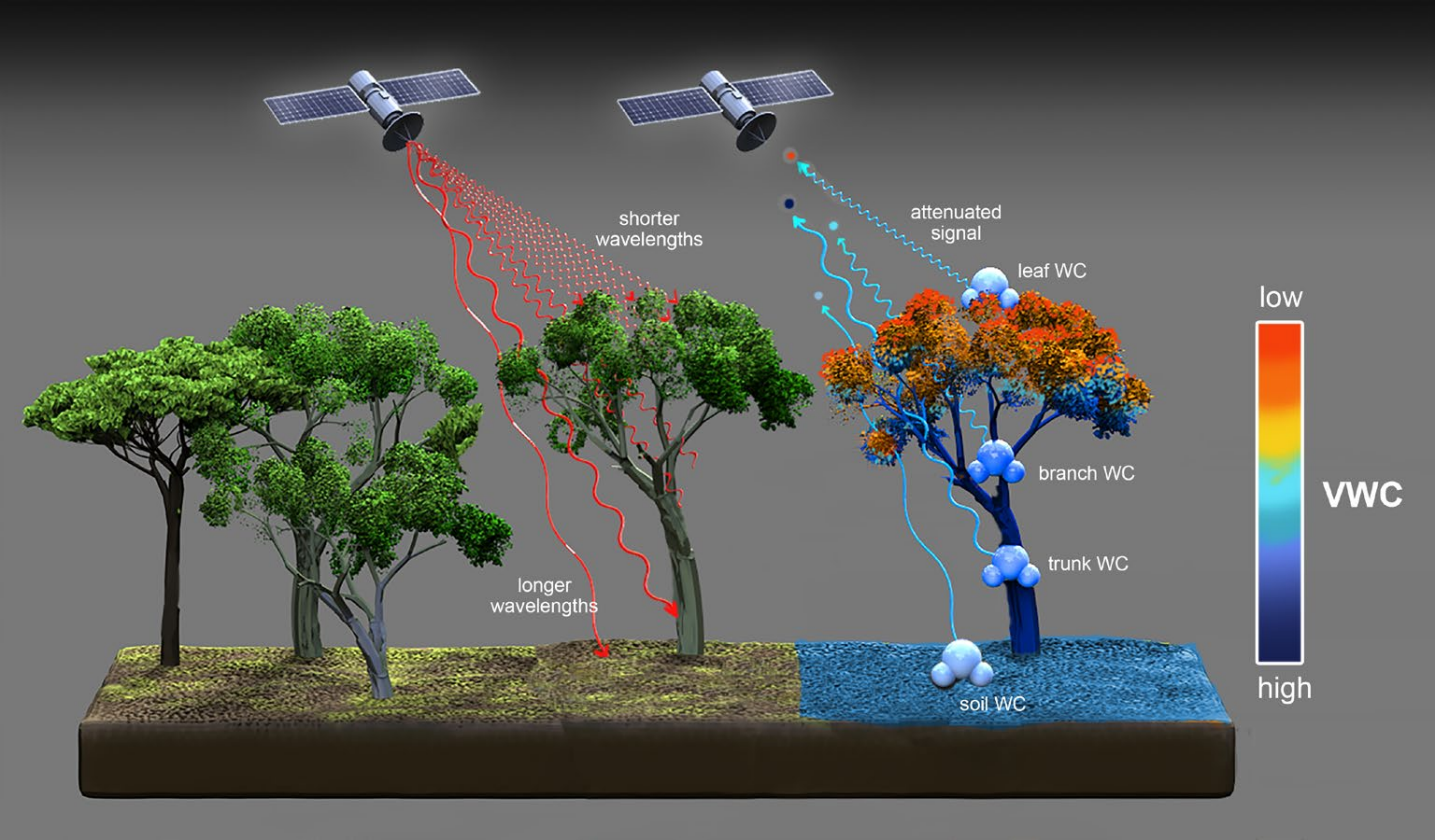
Microwave remote sensing is able to observe water content in forests. The canopy layers represented in each measurement (the penetration depth) varies across different microwave frequency bands (and thus different wavelengths), as show through different red and blue electromagnetic waves. Observations represent deeper areas of the canopy as wavelengths increase (and frequencies decrease) from Ku‐band across X‐, C‐, and L‐bands to P‐band. Higher frequencies are most sensitive to leaves and branches while lower frequencies also have increasing sensitivity to trunks and soils. Red waves represent transmissions on a radar system while blue waves represent the returns, with dots at the end of each wave representing different magnitude backscatter coefficient measurements depending on the water content (colorbar) of the different vegetation components each wavelength is sensitive to. If only the blue waves are considered and the dots are interpreted as measurements of VOD, the figure is representative of a radiometer system instead

Microwave remote sensing measurements respond directly to changes in VWC due to their sensitivity to the dielectric constant and thus to free water volume (i.e., water that is not chemically bound) in vegetation (including leaves, branches, stems) (Ulaby & El‐rayes, 1987). Depending on the electromagnetic frequency, the depth of penetration of microwaves into the forest canopy may vary (Figure 3). The sensitivity to VWC is expressed as the mass of water per ground area (i.e., in units of kg water/m^2^) (Schmugge & Jackson, 1992). The main observation of VWC from active and passive microwave remote sensing is through the vegetation optical depth (VOD), which is a measure of how much the VWC attenuates the microwave signal from the soil surface (Frappart et al., 2020; Konings et al., 2019). The theoretical basis for this relationship and typical retrieval approaches are reviewed extensively in Frappart et al. (2020).

Vegetation optical depth is a dimensionless quantity, with higher values indicating more attenuation and hence a larger quantity of VWC. VOD is often assumed to be linearly related to VWC (Jackson & Schmugge, 1991) with a coefficient depending on the frequency of observation, forest type, and structure (height, biomass density, and gap size). Studies comparing microwave sensing to in situ measurements of VWC have been able to establish the linear relation for a wide range of vegetation types (van Emmerik et al., 2015; Jackson & Schmugge, 1991; Sharma et al., 2020). Note that direct retrieval of VWC—rather than a quantity proportional to VWC—has not yet been performed at global scale. Nevertheless, the linear relationship between VOD and VWC has enabled a range of applications such as detection of water stress in forest ecosystems (Frolking et al., 2011; Rao et al., 2019; Saatchi et al., 2013), quantification of the diel cycle of VWC (van Emmerik et al., 2015; Konings, Yu, et al., 2017; Schroeder et al., 2016), and estimation of seasonal changes of VWC related to phenology (Tian et al., 2016; Wang et al., 2020; Xu et al., 2015).

### 3.2 Challenges and opportunities for estimation of VWC from remote sensing

While applications of microwave vegetation remote sensing are growing rapidly, some long‐standing challenges remain. At ecosystem‐scale resolutions, VWC depends not only on water stress but also on seasonal to interannual changes in biomass (Brandt et al., 2018; Konings et al., 2021; Liu et al., 2015). Changes in relative water content can be disentangled from changes in phenology and biomass by considering diel or other short‐term timescales, as further discussed in Section 4. Nevertheless, this complicates interpretation of VWC observations.

Because microwave remote sensing is sensitive to both VWC and soil moisture, retrieval algorithms are needed to separate these two factors. However, most operational retrieval algorithms represent the VWC as consisting of a homogenous cloud of water droplets, which neglects the roles of vertical and horizontal variations in water content, canopy gaps, surface water from dew and rainfall interception (Xu et al., 2021), etc. Not only do these simplifications risk incurring retrieval or interpretation errors for both existing datasets and future VOD retrievals, they also cause a missed opportunity. Because the sensitivity of microwave observables to these factors varies based on frequency and polarization (Baur et al., 2019), heterogeneity in water content across different heights in the canopy could in theory be accounted for. If overlapping observations at multiple electromagnetic frequencies are available, these could then be combined to determine water content across different heights in the canopy. However, doing so will require more sophisticated electromagnetic models (Saatchi & Moghaddam, 2000; Steele‐Dunne et al., 2017), which in turn require detailed information about tree and forest structure. Recent progress in remote sensing‐derived vegetation structure information may be able to help fill this gap (Dubayah et al., 2020; Quegan et al., 2019; Yu & Saatchi, 2016). A more mechanistic understanding of microwave observations will also offer more synergies with optical and spectroscopic methods (Bohn et al., 2019), which are most sensitive to the upper layers of the canopy and can therefore provide complementary information to deeper microwave observations. In the case of passive microwave observations, it may also enable better accounting for changes in temperature across the canopy, and associated improvements in retrieval accuracy (Parinussa et al., 2016). However, further development of more advanced retrieval approaches will require coordinated field campaigns for calibration and validation, including non‐destructive ground‐based samples of water content such as those in Section 2.3. If the relationship between water content and (leaf or xylem) water potential can be quantified (Section 5), existing water potential measurements—while sparse—could also be used in validation field campaigns. Indeed, given the sparsity of ecosystem VWC measurements, additional validation field campaigns would also be useful for validating existing VOD retrieval methods.

Lastly, existing satellite observations of VOD from radiometers (Du et al., 2017; Konings et al., 2017; Moesinger et al., 2020; Wigneron et al., 2021) and scatterometers (Frolking et al., 2006) can be noisy when only individual measurements are considered. Furthermore, they have coarse spatial resolution (25–50 km) and cannot reliably separate changes of VWC from other disturbance and recovery processes associated with canopy cover and biomass. The applications of these measurements will improve substantially if the spatial resolution of the observations reaches the landscape scale (100–1000 m) (Martínez‐Vilalta & Lloret, 2016).

### 3.3 Past and future sensors for VWC observations

Despite the challenges above, our ability to monitor VWC dynamics with microwave remote sensing is currently constrained by sensor availability, not by technology. Existing and planned passive microwave observations (e.g., AMSR‐E, SMAP, and CIMR) and scatterometers (e.g., QuikScat and ASCAT) at different electromagnetic frequencies provide long‐term coarse resolution observations to monitor soil and vegetation water status regionally, while less frequent but high‐resolution synthetic aperture radar measurements (e.g., Sentinel‐1 (Torres et al., 2012), NISAR (Kumar et al., 2016)) help to quantifying landscape‐scale variations of VWC. However, the greatest limitation of existing spaceborne measurements from sun‐synchronous orbits is the lack of diel observations of VWC that can be directly linked to the plant physiology and to water and carbon exchange. At such timescales, there is minimal influence from changes in phenology and forest structure on the total ecosystem‐scale VWC (Section 4). Microwave observations from RapidScat onboard the International Space Station (van Emmerik et al., 2017; Konings, Yu, et al., 2017) and from ground‐based tower systems (Holtzman et al., 2021; Monteith & Ulander, 2018; Schneebeli et al., 2011; Vermunt et al., 2020) have demonstrated the feasibility of quantifying the VWC dynamics throughout the day. Thus, the ability to monitor the diel signal of VWC is driven by the orbital choices of the existing sensors, not by limitations of the microwave observations’ intrinsic sensitivity. Section 4 further discusses the ways different timescales of analysis enable study of different aspects of forest drought response.

## 4 DERIVING PROCESS UNDERSTANDING FROM VWC

### 4.1 VWC information depends on analysis timescales

**FIGURE 4:**
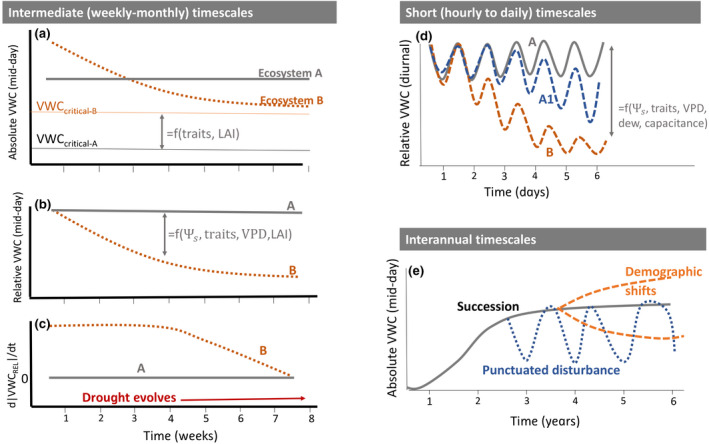
What can vegetation water content tell us about plant stress? The absolute value of VWC (panel a, shown as mid‐day values) is difficult to interpret without context about its maximum and critically limiting values (e.g., the VWCcritical). For example, while Ecosystem B initially has higher absolute VWC than Ecosystem A, its VWCcritica is also higher. When VWC is expressed as a relative value compared to the seasonal maximum (panel b), Ecosystem B emerges as consistently more stressed than A, with the difference between the two reflecting traits, structure, as well as environmental states (soil water potential, VPD). The time derivative of relative VWC (panel C) illustrates that the time change in VWC can be zero for both very stressed and very unstressed ecosystems, but the change in d|VWC|/dt over periods of weeks to months is highly informative of the ecosystem water status. On the right side, panel (d) shows differences in the diurnal amplitude of relative VWC for ecosystems experiencing little stress (A), intermediate (A1), and more severe stress (B). Panel (e) shows long‐term (interannual) changes in absolute VWC attributable to succession, disturbance, and demographic shifts

Remote measurements of VWC can extend process‐level understanding in forests by leveraging variation in VWC in space and time. VWC integrates processes associated with water storage and fluxes of different forest water reservoirs (leaf, wood, soil) at different timescales. We therefore posit that measuring VWC dynamics can be crucial for understanding, quantifying, and modeling ecological and hydrological processes at roughly three timescales (Figure 4).

At the timescale of multiple years to decades (Figure 4e), VWC patterns largely reflect forest biomass and structure (Konings et al., 2019; Liu et al., 2013, 2015). Thus, spatial patterns in VWC can shed light on variation in biomass, canopy structure, biome boundaries, and species‐level traits that influence water content (e.g., wood density (Gentine et al., 2016; Araújo et al., 1999)). The sensitivity of VWC to aboveground biomass can inform disturbance and land‐use change dynamics (Liu et al., 2015; Pugh et al., 2019) and potentially slower, non‐disturbance shifts in demographic rates such as succession or climate‐driven increases in mortality that lead to changes in species composition (Anderegg et al., 2020; Trugman et al., 2020; van Mantgem et al., 2009). Multi‐year averaged measurements of VWC can therefore provide a powerful set of measurements for spatial scaling and quantifying ecological dynamics—particularly those related to biomass, rather than water content or physiology alone per se—at biome, continent, and global scales.

At the timescale of multiple weeks to months (Figure 4a‐c), VWC dynamics may reflect progressive dehydration of multiple tissues of trees associated with changes in soil water potential and xylem function, as well as changes in forest leaf area index caused by leaf shedding or leaf flushing (Frolking et al., 2011; Jones et al., 2011; Konings et al., 2019; Tian et al., 2017). Assuming a threshold‐type response (Section 2.2), VWC may therefore be useful to assess the risk of drought‐induced forest mortality and flammability risk. The slope of VWC curves during prolonged droughts and after post‐drought precipitation events may also be used as metrics to compare community‐level drought resistance and recovery capacity (Asefi‐Najafabady & Saatchi, 2013; Suding & Hobbs, 2009). More gradual slopes likely means that the tree cover has more mechanisms that minimize dehydration, indicating higher resistance.

At diel timescales (Figure 4d), changes in biomass are slow and VWC dynamics reflect the balance of transpiration and root water uptake, as well as redistribution of water through trees (van Emmerik et al., 2017; Konings, Yu, et al., 2017). These timescales are thus most closely affected by root, xylem, and stomatal responses to drying soil and air. As a result, VWC observations at diel timescales are arguably best able to isolate the effects of water stress. VWC variations across the diel cycle are also closely related to belowground processes, which are difficult to disentangle at other timescales (see Section 6). The shape of the diel cycle of VWC can be used to detect water stress (Nelson et al., 2018), before it is detectable through other leaf properties. Therefore, the diel dynamics of VWC also hold promise as an early warning signal for forest risks.

### 4.2 Complementary measurements to improve VWC interpretation

By combining VWC measurements with complementary field and remote sensing data, we can vastly improve process‐level understanding in forests across multiple timescales. At the timescales of multiple years to decades, data on species composition, forest structure and biomass, demography, and disturbance history could be used to test for species‐, age‐, or disturbance‐dependent responses to drought in forests (Hanson & Weltzin, 2000; Zhang et al., 2018). Disentangling the sources of within‐ and cross‐ecosystem variability in patterns of VWC is facilitated by observations of factors such as soils and topography and canopy structural attributes like leaf area index and vertical architecture. At the intermediate timescales of multiple weeks to a month, soil moisture and meteorological conditions and phenology are the major constraints on VWC dynamics. Additional measurements such as volumetric soil moisture, temperature, precipitation, and vapor pressure deficit, along with phenology‐related data, can provide crucial context. At diel timescales, complementary measurements related to canopy composition and biomass dynamics are less necessary and less useful. Surface soil moisture dynamics and evapotranspiration estimates (or more directly, thermal imagery sensitive to evapotranspiration) may provide complementary information about the water distribution through the soil‐plant‐atmosphere continuum at remote sensing scales. Observations and estimates of canopy water content from dew or rainfall interception will also be useful, to remove these signals from the observed VWC proxies (Binks et al., 2021; Vermunt et al., 2020; Xu et al., 2021). Additionally, analyses based on diel variations in VWC may be constrained based on functional trait data (i.e., hydraulic traits), where available (Trugman et al., 2020).

Beyond complementary datasets, process models are a necessary counterpart to VWC measurements to enable interpretation of VWC patterns and its underlying mechanisms (Xu et al., 2021). Models can also benefit from the constraints that VWC can place upon their interpretation, and thus model‐measurement integration is a win‐win situation. The type of models that can integrate VWC information most effectively are hydraulically enabled (e.g., Christoffersen et al., 2016; Kennedy et al., 2019; Li et al., 2020; Mencuccini et al., 2019), and would be able to simulate water pools (Martinez‐Vilalta et al., 2019). Such models can operate over a range of spatial scales and on timescales that span minutes to years, enabling process understanding of VWC over short to long timescales. Examples of model‐data benefits include the opportunity to examine the role of community‐scale plant water storage and capacitance (simulated via models) in regulating the observed VWC variation, understanding how transpiration rates may drive variation in observed VWC, or understanding belowground controls on water uptake (as further discussed in Section 6). To link models and data, an ecosystem‐scale water release curve could be generated in which VWC is the dependent variable and simulated community‐scale water potential is the independent variable. Such curves are further discussed in the next section.

## 5 SCALING WATER CONTENT TO ECOSYSTEM SCALE

### 5.1 An ecosystem‐scale pressure–volume curve

In order to use VWC as a constraint on plant hydraulic models that simulate the dynamics of water potential (Ψ), the relationship between Ψ and VWC must be known at the ecosystem scale. As a thermodynamic property of the water itself (i.e., its free energy), the Ψ can be averaged at any scale and across media, enabling the consideration of an instantaneous Ψ for a cell, a leaf, a shoot, a branch, or a tree (Pallardy et al., 1991; Scholander et al., 1964), or potentially, the whole ecosystem. Given the spatial scale of remote sensing observations, estimating Ψ from orbit would require ecosystem‐scale Ψ‐WC curves (eco Ψ‐WC; pronounced, *ecopsych*). Such curves can be conceived as large‐scale analogs of tissue‐scale P‐V curves: graphical plots of the relationship between relative or absolute water content and Ψ, commonly constructed by dehydrating leaves or stems (Richter, 1978; Scholander et al., 1964; Tyree & Hammel, 1972). They are also analogous to soil water retention curves (Hillel 2013). The earliest conceptualization of a P‐V curve was applied to twigs and leaves, and recognized the potentially large variability in water retention properties of the constituent living cells, but showed that cellular‐level P‐V curves follow a remarkably similar form to that of the bulk P‐V curve (Tyree & Hammel, 1972). We argue that the scale jump from organ‐level P‐V curves to the canopy is no greater than from cells to organs, and as such, the eco Ψ‐WC concept is more than possible, it is inevitable.

**FIGURE 5:**
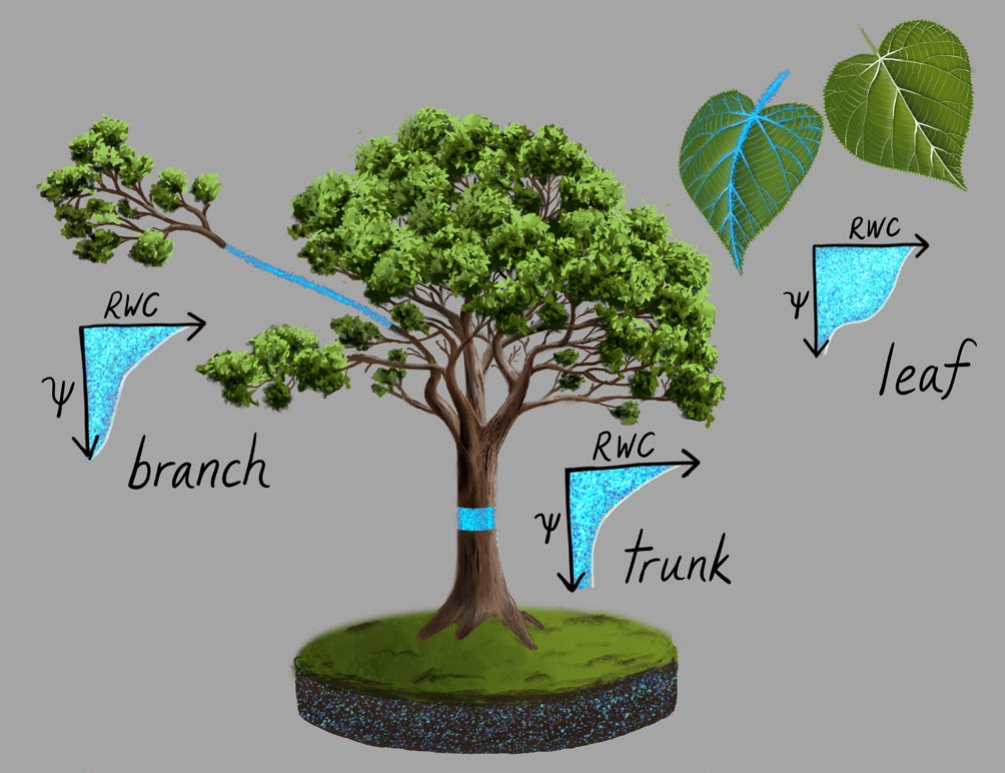
Vertical distributions of tissue‐specific water retention properties (RWC – Ψ curves), biomass, and sensor penetration depth all jointly determine remotely sensed water content and its temporal variation. Several hypothesized curves delineating gradients of capacitance, defined as the change in relative water content relative to that of water potential (C = ΔRWC/ΔΨ) are shown. Therefore, temporal variation in remotely sensed metrics of VWC will be determined not only by temporal variation in Ψ, but by potentially large differences in the exchangeability of water in response to changes in Ψ across different plant tissues, and the response of sensor penetration depth to changes in water content

Notably, even for one ecosystem at any moment in time, the eco Ψ‐WC would consist of a family of curves, each dependent on the spatial scale being used to average Ψ and VWC, and on fluxes through the system that influence the relative distribution of water among individual trees, and within trees, among cells and organs. When modeled comprehensively and given enough spatial resolution, the eco Ψ‐WC could enable a full three‐dimensional suite of Ψ‐WC relationships, at a range of scales (per leaf area, ground area, volume, or mass; by canopy layers, plant organs, sizes or species; scanning layers vertically vs. integrating across volume). Across the suite of possible eco Ψ‐WC curves, some may be especially powerful for particular applications, which there are potentially a wide range of (Table 1, see also Section 2). Indeed, the fine‐scale distribution of canopy VWC and Ψ gained from eco Ψ‐WC curves would allow assessing (1) the allocation of water throughout the forest including shifts in storage (e.g., wood swelling (Pfautsch et al., 2015)); (2) water status thresholds for loss of function throughout the ecosystem (Martinez‐Vilalta et al., 2019; Sack et al., 2018; Trueba et al., 2007), (3) the driving forces for water movement; and (4), with knowledge of hydraulic conductances and capacitances, the water flows throughout the ecosystem (Figure 5). Furthermore, there is potential to extract parameters from eco Ψ‐WC curves, analogous to those extracted from leaf P‐V curves, to enable the consideration of how whole ecosystem drought resilience and its determinants shift over the course of the day and seasonally, and how ecosystem‐level drought responses vary across ecosystems of different diversity, climate, or soil type.

**TABLE 1:** Applications of remotely sensed vegetation water content, relative water content at ecosystem scale (VWC_eco_, normalized by its maximum value), water potential (Ψ), and the ecosystem Ψ‐WC curve (eco Ψ‐WC)

VWC	VWC_eco_	Ψ	Eco Ψ‐WC
· Estimation of water distribution throughout the ecosystem and its dynamics with time, and environmental change · Can be directly converted to an ecosystem‐scale live fuel moisture content for fire risk estimation, by dividing VWC by aboveground dry biomass. · VWC may also predict drought‐induced mortality in trees	·Thresholds for stomatal control, photosynthesis, wood growth, embolism, hydraulic dysfunction, mortality, etc., for a given tree or tissue and potentially ecosystems.	·Thresholds for stomatal control, photosynthesis, wood growth, embolism, hydraulic dysfunction, mortality, etc., for a given tree or tissue and potentially ecosystems ·Overall driving forces for water flows at landscape, community, and ecosystem scale · Additional signal regarding tissues and belowground soil water potential ·Given known hydraulic conductances and capacitances, estimates of flows through given components of the system at any scale	· Scaling up phenomena from cells to organs, to plants, to ecosystem · Determination of water allocation throughout the forest including shifts in storage · Transfer function for data assimilation of remotely sensed VWC or VWC validation campaigns · Clarification of the most informative water status thresholds for loss of function throughout the ecosystem · May yield ecosystem Ψ‐WC parameters useful for comparative assessment of drought tolerance and water relations across space and time

The time of day considered affects the utility of eco Ψ‐WC curves. At predawn and midday or early afternoon, they may provide especially informative snapshots. Eco Ψ‐WC curves estimated at predawn have the advantages of simplicity and stability because, at equilibrium, flows through the system will not affect the distribution of water, and the root‐zone soil Ψ will also be indicated. Predawn Ψ may best yield certain thresholds for determining ecosystem function (e.g., wood growth, plant maximum hydraulic conductance) (Cabon et al., 2020). By contrast, midday or early afternoon eco Ψ‐WC curves include the near minimum Ψ and WC values, corresponding to the strongest diel drought stress (with the exact time of greatest stress variable depending on ecosystem type and meteorological conditions). Because the ways in which eco Ψ‐WC curves can be used vary by time of day, diurnally variable observations of VWC may be particularly useful for forest drought stress studies analyzing ecosystem‐scale Ψ variations.

### 5.2 Determining eco Ψ‐WC curves

To derive the eco Ψ‐WC, one must apply a modeling approach to the P‐V curves of tree tissues at given times (considering each tissue's water storage capacity and elastic properties), and scale these up based on forest structure (tree volume; tree sizes; allometries for roots, stems, and leaves; water content distribution) (Figure 5). Furthermore, the model must be dynamic, as Ψ will depend on the flow rate and hydraulic conductances and capacitances throughout the soil‐plant‐atmosphere continuum. While VWC is usually measured as the mass of water in vegetation per unit ground area, the relative water content (RWC), which normalizes tissue water content by the maximum (i.e., turgid) water‐holding capacity of vegetation, better allows comparisons across organs, species, growth stages, and scales, and may thus be the more useful quantity for use in an eco Ψ‐WC curve. To determine relative water content from absolute water content, a saturation water content must be estimated. One approach to measuring canopy RWC is to normalize VWC by its annual or seasonal maximum (resulting in a relative vegetation water content at ecosystem‐scale, VWC_eco_; Rao et al., 2019) while accounting for changes in aboveground biomass, for example, from leaf abscission during a drought event. This accounting requires ancillary information on phenology. It is also important to consider whether leaf surface water is included in the eco Ψ‐WC curve, or separated during the remote sensing retrieval of VWC (see Section 3.2). Along with tissue‐level scaling (including for components such as bark or roots, for which P‐V curves may not be as readily available as for leaves, see Section 2.3), information about canopy structure and diversity is needed to scale from trees to ecosystems. Structure and diversity information may be derived from a combination of forest inventories and remote sensing, including from ground, airborne, or space‐based lidar.

In order to relate eco Ψ‐WC curves to microwave retrievals of VWC, we need information on the vertical structure of the canopy and the frequency‐dependent penetration depth of VWC observations (Figure 4e–h). Because, at a given electromagnetic frequency, microwaves also pass farther through canopies with less water (Section 3.1), the effective depth that a given VWC measurement represents is likely to vary in space and time, particularly during a drought. To account for penetration depth variations, different eco Ψ‐WC curves be built to apply to specific electromagnetic frequencies. Once created, eco Ψ‐WC curves should then enable linking remotely sensed VWC to models, leading to improved quantification of plant traits, belowground variables (as further discussed below), and other factors affecting ecosystem drought response.

## 6 INFERRING BELOWGROUND ACTIVITY FROM ABOVEGROUND VWC OBSERVATIONS

**FIGURE 6:**
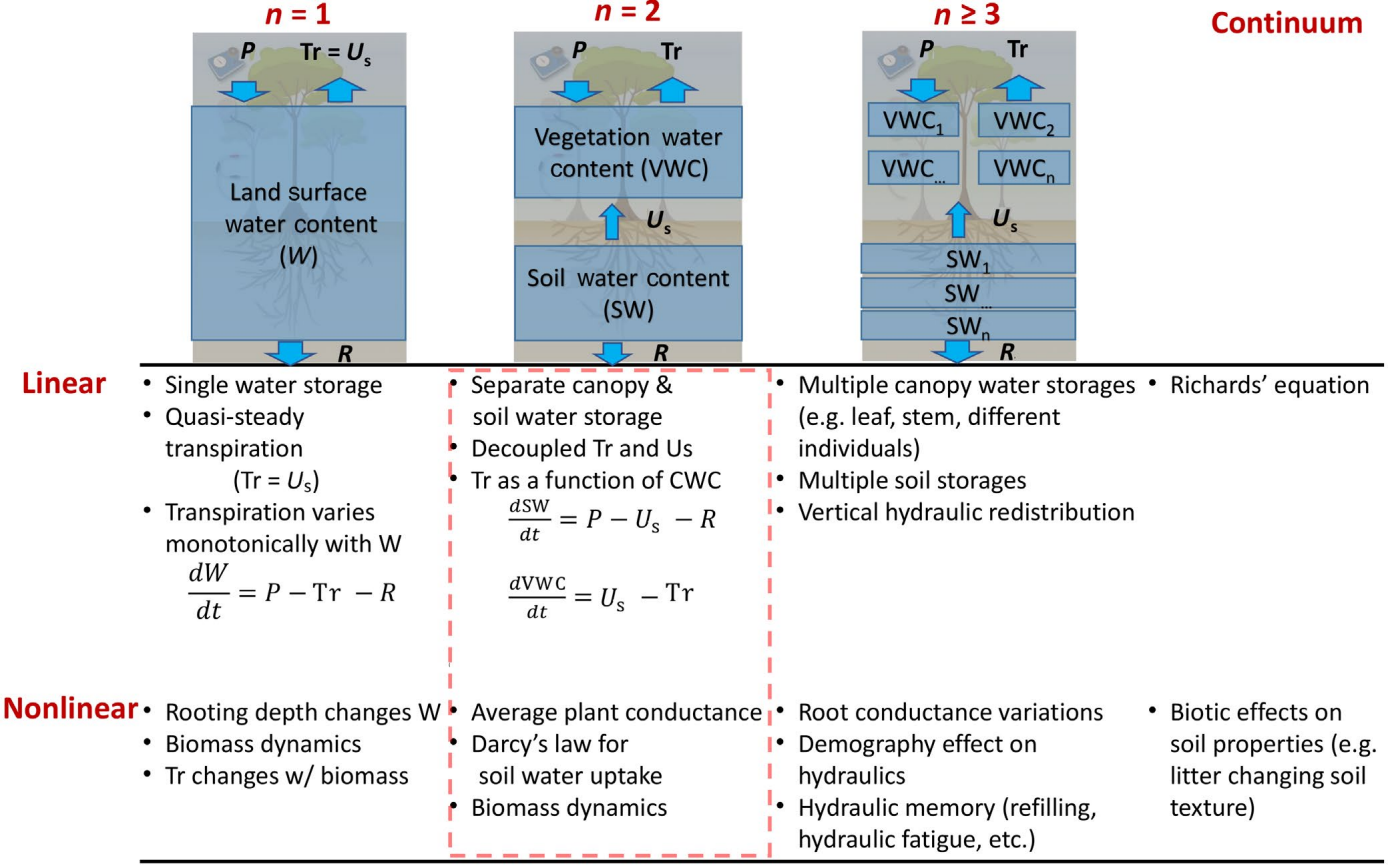
Conceptual diagram showing the complexity of vegetation–soil water dynamics viewed in the dimensionality–nonlinearity plane. The red dashed box shows the near‐term research direction for inferring belowground dynamics enabled by new observations of VWC from remote sensing. A diurnal hysteresis between VWC and soil water cannot be captured with traditional ecohydrological models that consider a single land surface water pool (n=1) and can only be explained with a two (or greater) pool framework (n≥2), which further allows for inference of vegetation water uptake based on the timing and magnitude of the hysteresis. Figure adapted from Strogatz (2015)

A complete description of forest responses to drought requires accounting for several belowground factors, including the regulation of root water uptake, its three‐dimensional distribution, or competition among differing rooting systems, among others (Manoli et al., 2017). However, remote sensing measurements are currently unable to measure soil water or water fluxes in the root‐zone directly. Instead, belowground conditions must be inferred from aboveground information, such as VWC observations. This can be achieved through an inverse approach based on the analysis of aboveground conditions linked to belowground processes. When doing so, the complexity of the belowground mechanisms that are accounted for can span a wide range (Figure 6). Conventional ecohydrological models that seek to infer belowground conditions without considering VWC (assuming a single land surface water pool, e.g., n=1 in Figure 6) implicitly consider root water uptake to be in balance with transpiration (Chitra‐Tarak et al., 2018; Dralle et al., 2020; Fan et al., 2017; Kleidon, 2004), and therefore cannot resolve root water uptake variations that deviate from transpirational demand (Chuang et al., 2006; Hollinger et al., 1994; Phillips et al., 1997). Even non‐linear models that account for, for example, rooting depth changes with soil water content or the influence of biomass are not able to separate out root water uptake from transpiration if they do not account for VWC. We therefore argue that accounting for VWC (n ≥ 2 in Figure 6) is a necessary first step in inferring belowground conditions. If VWC observations across different vegetation layers are available (n ≥ 3 in Figure 6), this will enable greater detail in the inferred belowground conditions, such as soil water content variations across different depth layers and hydraulic redistribution.

Inferring belowground activity from VWC dynamics requires solving the complex interactions between water pools and water fluxes. One approach for doing so relies on building plant hydraulic models and using data assimilation or optimization methods to constrain the parameters and states of these models (Liu et al., 2020; Mirfenderesgi et al., 2016). As with any data assimilation/optimization method, innovations in the assimilation/optimization technique and cost function specification (i.e., which mathematical function is optimized) may further improve the ability to accurately make belowground inferences (Dietze et al., 2011; Trudinger et al., 2007). Ensuring a reasonable balance between model parsimony (e.g., making sure the number of degrees of freedom of the model is not much greater than that of the observations) and model complexity (to ensure realistic dynamics can be captured) is also key for accurate assimilation methods. Ultimately, the accuracy of such inferences likely inherently depends on the relative sensitivity and information content of different observations, including the VWC estimates. Nevertheless, some early applications of data assimilation with remotely sensed VWC estimates show this approach has promise (Liu et al., 2021; Liu et al., 2021). In each of these studies, observations at two times a day were used, but a more complete diel cycle may act as an even stronger constraint.

**FIGURE 7:**
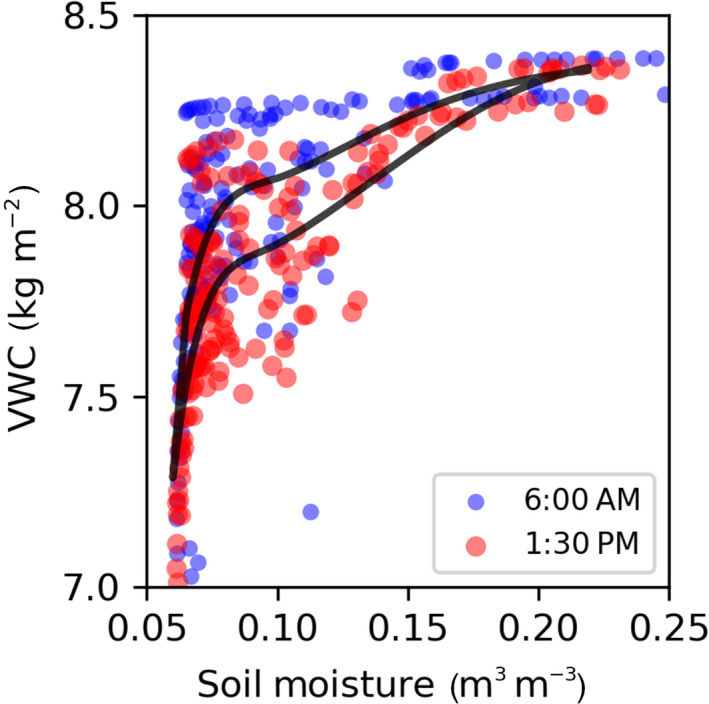
Example phase diagram of simulated dynamics of VWC and root‐zone soil moisture content for a model test bed site in an Amazon moist forest using a hydraulics‐enabled terrestrial biosphere model (ED‐2.2‐hydro, Xu et al., 2021). The diurnal hysteresis (closed curves in black between VWC and soil water cannot be captured with traditional ecohydrological models that consider a single land surface water pool (n=1 in Figure 6). Such hysteresis can only be explained with a two(or more)‐pool framework (n>=2 in Figure 6), which further allows for inference of vegetation water uptake based on the timing and magnitude of the hysteresis

As an alternative to computationally expensive data assimilation methods, additional information may be gained by considering the phase dynamics of soil and VWC (Figure 6). At any given time, the evolution of both soil and VWC is influenced by sap flow, plant water storage (in particular VWC) and other factors (i.e., precipitation, transpirational losses). The sap flow, in turn, depends on both the soil and vegetation water content (both through the potential gradient between each, and through VWC's influence on xylem conductance). Thus, for each of soil water content and VWC, the evolution of one water pool depends on the value of the other pool at any given amount of time. This state dependence inherently generates a hysteresis across diel timescales, as illustrated in Figure 7 (Lin et al., 2019; Zhang et al., 2014). With increasing data availability of VWC and (surface) soil water dynamics from remote sensing products, analysis of this hysteresis and the phase dynamics more generally can be used for detecting lags and tipping points. A specific example of such a phase dynamical analysis is mathematically illustrated through an analogy with the much‐studied predator–prey (also known as Lotka–Volterra) ecological model (Wangersky, 1978) in the Data S1 (where VWC is the predator in the Lotka–Volterra analogy, which preys on root‐zone water content). Development of simplified mathematical models such as these will enable more sophisticated phase dynamics interpretations using VWC or even multi‐layer VWC datasets, if those become available (Section 3.2). Taken together in a suite of work with data assimilation and inference approaches, VWC analyses can therefore generate significant progress in determining belowground hydrological activity with remote sensing at global scale.

## 7 THE NEED FOR SPACEBORNE DIEL OBSERVATIONS

In this section, we reflect on the value of current satellite measurements of VWC and then consider what satellite observational strategy might be most useful for understanding how forest ecosystems respond to drought in a changing climate. As discussed in Section 3, existing observations of VWC from space are mainly based on opportunistic analyses of measurements originally designed and used for other science applications. These datasets have been used for many studies of forest drought responses. However, they are all limited by a particular set of measurement characteristics. For example, most of these satellites are in sun‐synchronous orbits, which results in an overpass at each location on earth at a consistent time of the day, which is usually around 6:00 AM and PM or around 1:30 AM and PM, depending on the sensor. The sensor revisits every location on earth every 2–3 days, but only two particular times of day are ever observed. This prevents a full view of the diurnal cycle. In terms of spatial scale, available microwave remote sensing datasets of VWC exist at a range of resolutions (tens of meters to tens of kilometers), depending on the sensor type (radiometers, scatterometer, synthetic aperture radar (SAR)). However, a trade‐off exists. Relatively coarser datasets from radiometers and scatterometers have spatial resolutions of tens of kilometers, but are designed such that they revisit each location every few (2–3) days. By contrast, SAR sensors are able to obtain observations at scales of tens of meters, but only revisit a given location infrequently, often in an irregular fashion and usually averaging only a few (1–3) observations per month in any given location. Some sensor combinations provide observations more frequently, but only over small areas (e.g., Sentinel‐1 over Europe (Torres et al., 2012)). Furthermore, not all SAR instruments measure multiple polarizations in all observing modes. Measurements of different polarizations are required to disentangle the contributions of soil moisture and vegetation to observed backscatter. Additional polarizations may also further be useful to separate VWC from vegetation structural changes. Although VWC has been successfully retrieved from different combinations of possible polarizations (e.g., VV and VH from Sentinel‐1 (Han et al., 2019; Rao et al., 2020; Vreugdenhil et al., 2018), or VV and HH from QuikScat (Oveisgharan et al., 2018)), more research is needed to better understand the relative value of different polarizations and the optimal design of retrieval algorithms.

We recommend, at a high level, alternative satellite observations that can address several scientific measurement requirements to detect forest water stress and response to droughts. First, to predict how transpiration responds to droughts, it is necessary to quantify the influence of vegetation water content on stomatal conductance. This requires measurements of VWC throughout the day because the time of maximum vegetation water stress varies significantly from day to day, and because the shape of the diurnal cycle of VWC allows differentiation between the effects of limitations in root water uptake, transpiration, and redistribution of water storages through the canopy. Therefore, frequent observations of VWC throughout the day, at least every few hours, will allow for quantifying the response of plant stomatal conductance to water stress. Moreover, as discussed in Section 2.2, tree mortality and forest flammability have been shown in a few studies to have a threshold‐like response to declines in VWC. Whether such threshold‐like behavior holds at ecosystem scale and how such thresholds vary across biomes is unknown. Again, observations of the diel cycle will ensure that the periods of maximum VWC stress—where VWC is most likely to decrease below any thresholds—are determined. Beyond capturing the diel cycle, the revisit times between days of observations should also be sufficiently small so as to ensure periods of maximum stress are observed. Additionally, the temporal observations of VWC must extend across seasonal cycles of water availability to allow separation of the impacts of long‐term droughts from climatological seasonality, and must include multiple years of observations to capture both episodic climate extremes, interannual variability of forest canopy dynamics, and long‐term gradual climate stress. A long enough interannual observational record across large (e.g., continental to global) scales would enable determination of the factors driving how well and how fast biomes adapt to shifts in climate and seasonality.

The needs identified above—for diel observations with frequent revisit and over an extended period of time—suggest that a geostationary platform (rather than the typical sun‐synchronous orbits previously used for microwave satellites) would be needed to better quantify forest responses to drought. Furthermore, for each of the above applications, observational datasets would be particularly useful if they were able to distinguish landscape‐scale (e.g., kilometer‐scale) spatial variations, to allow detection of landscape‐scale variations of forest ecosystem response to water stress that depend on edaphic conditions, topography, forest structure, and land use history. At the high altitude of geostationary orbits, passive microwave radiometer systems cannot provide the required spatial resolutions. However, recent developments in radar technology, particularly at X‐ and Ku‐band frequencies (1–3 cm wavelength) provide an excellent opportunity for relatively high‐spatial resolution observations from a geostationary platform (Rodríguez et al., 2019; Xiao et al., 2020). Alternatively, a collection of smallsats (cubesats or other similar size classes) with different daily observation times could also potentially achieve the required temporal and spatial resolution (e.g., the Capella Space constellation (Stringham et al., 2019)). Each individual satellite could be placed in a sun‐synchronous orbit, but combining observations from multiple instruments would still enable observations of the full diurnal cycle. This approach has been successfully used to increase observational frequency by the Cyclone Global Navigation Satellite System (CYGNSS) (Ruf et al., 2019). Given the multiple potential approaches for developing spaceborne observations of VWC at diurnal cycles (although note the remaining technical challenges associated with retrieval algorithms, see Section 3.2), further research is needed to determine the best technological solution. Using the relatively high X‐band or Ku‐band frequencies will enable higher spatial resolution and reduce the effect of soil moisture on the radar backscatter observations, increasing retrieval accuracy. RapidScat radar observations at Ku‐band have previously been shown to successfully capture diel dynamics of VWC (van Emmerik et al., 2017; Konings, Yu, et al., 2017). Furthermore, at X‐band, cross‐comparison with existing sun‐synchronous datasets at times of near overlap (Du et al., 2017; Moesinger et al., 2020) could be used for calibration of the VWC retrieval algorithm. If technologically feasible, multiple electromagnetic frequencies could also be combined in a single observing platform to enable determination of VWC across multiple layers of the canopy (Section 3.2). We note that a geostationary platform would not be able to observe the entire globe, but continental coverage can nevertheless probe forest behavior across a range of biomes. A focus on the Americas may be particularly useful given the diversity of biomes and vegetation types spanned by this region and the relatively larger number of available field measurements compared to many other regions. Diel microwave observations of the Americas could also benefit from existing and future geostationary measurements covering the same area, such as solar‐induced fluorescence from the Geostationary Carbon Cycle Observatory (GeoCARB, Moore et al., 2018) and land surface temperature from the Geostationary Operational Environmental Satellites (Khan et al., 2021). A satellite mission with the above characteristics could be used not only to address the specific hypotheses discussed in the previous paragraph, but could also be used for a number of operational applications, such as predicting crop yields in the face of water stress, or improving fire risk models. The satellite design proposals above are summarized in Table 2.

**TABLE 2:** Relationship between science and application goals and instrument functional requirements (as driven by the measurement requirements and science and application objectives necessary to meet the science and application goals) for a proposed set of new satellite observations

Science and application goals	Science and applications hypotheses	Measurement requirements		Instrument functional requirements
**Science question:** How do forest ecosystems respond to droughts in a changing climate?	There is a water content threshold beyond which tree mortality and flammability increase and productivity decline	**Science requirements**		
		Landscape‐scale VWC of forest ecosystems at 1σ < 1‐kg/m^2^ accuracy	Radar reflectivity at spatial resolutions of 1–3 km	X‐band, Ku‐band, or multiple frequency (Ku‐ & L‐band) scatterometer or SAR Multiple polarization (HH, VV, HV) geostationary platform or collection of smallsats that provides observations several times a day Large swath to cover North and South Americas (50^o^N ‐ 50^o^S) at 1–3 day repeat cycle
	Major resistance to water flux in forests is determined by changes in top‐canopy water content and its link to available soil water.	Diel changes of VWC at relative accuracy of 1σ < 10%	Radar reflectivity during day and night at multiple times throughout the day	
	Available soil water and the atmospheric environment will drive how well and how fast biomes adapt to climate change and shifts in seasonality	Seasonal changes of VWC at 1σ < 10% relative accuracy	Radar reflectivity at 1–3 day repeat cycle over minimum 3–5 years	
**Application goal:** Forecasting wildfires in forests and impacts of droughts on agriculture systems	VWC determines fire fuel risk and drought resilience of crops	**Application requirements**		
		Daily to interstorm changes of VWC at 1σ < 10% relative accuracy	Radar reflectivity at 1–3 km spatial resolution	X‐ or Ku‐band Multiple polarizations (HH, VV, HV) 1–3 day repeat cycle < 1‐km spatial resolution

## 8 SUMMARY

We described the potential benefits of spatially extensive and frequent microwave remote sensing‐based VWC measurements for studying forest responses to drought, including for prediction of mortality and fire risk. Although such data have been increasingly used to characterize forests and their water relations, we identified several technical and scientific developments which could significantly accelerate the utility of these data. Specific recommendations include:

1. Analysis methods that consider VWC at different timescales depending on the ecosystem process of interest: multiple years to decades for forest biomass and structure, multiple weeks to months for changes in leaf area and multi‐day trends of relative water content, and diel for changes in relative water content due to plant water uptake, redistribution, and loss.
2. Development of Ψ‐WC curves analogous to branch‐scale pressure–volume curves, to relate ecosystem‐scale VWC to an effective canopy‐scale water potential
3. Data assimilation and optimization methods that can use integrate VWC into plant hydraulic models for determination of xylem and stomatal traits as well as belowground activity
4. Investigation of phase dynamics for characterization of belowground activity
5. Development of retrieval algorithms that account for surface water and vertical variations of VWC within the canopy, rather than retrieving only the vertically integrated, average water content.

The approaches above should greatly accelerate the use of VWC for forest drought responses studies even with existing datasets. Nevertheless, to make the most progress, additional field campaigns are necessary for validating (multi‐layer) VWC retrieval algorithms in a wide range of data types, for testing eco Ψ‐WC curves, and for improved understanding of how VWC‐based thresholds for fluxes, mortality, and fire risk scale across the ecosystem. Finally, a new geostationary mission concept providing diurnally variable measurements of VWC, which can be integrated with existing measurements, would dramatically expand the scope of available forest drought response studies.

## CONFLICT OF INTEREST

The authors declare no conflicts of interest.

## Supporting information

Data S1

## Data Availability

QuikScat data shown in Figure 2 are available from the NASA PODAAC at https://podaac.jpl.nasa.gov/QuikSCAT.
