# Supplementary material for: Detecting forest response to droughts with global observations of vegetation water content
Source: Glob Chang Biol. 2021 Sep 25;27(23):6005–24. doi: 10.1111/gcb.15872 (PMC9293345; doi:10.1111/gcb.15872)
Supplement: Supplementary file 1 — Data S1 [file GCB-27-6005-s001.pdf]

Supplementary Information for "Detecting Forest Response to Droughts with Global Observations of Vegetation Water Content"

Analogy between predator-prey models and forest ecohydrologic systems

To illustrate the ‘genesis’ of hysteresis between VWC (labeled V for notational convenience) and root-zone water content (W), a ‘truncated’ predator-prey analogy is used (Mrad et al., 2020), also known as the Lotka-Voltera model (Wangersky, 1978). Phenomenologically, V ‘preys’ on W. The ‘prey’ W is intermittently recharged by throughfall and stem flow, both assumed to be proportional to rainfall (P) using a proportionality constant α that depends on a plethora of factors including leaf area index. In this naïve system, the main water supply pool for the plant water pool is the soil water in the rooting zone and the main sink is the atmosphere (through leaf transpiration T_r from all the leaves). A predator-prey analogy that simplifies all the aggregate effects of 3-D water transport in the soil-plant system yet preserves elements of their interactive effects leads to the following set of dynamical equations:

$$\frac{dW}{dt} = -aWV + \alpha P \qquad \text{(Eq. S1)}$$

$$\frac{dV}{dt} = +bWV - T_r, \qquad \text{(Eq. S2)}$$

where a and b are constants to be determined. A trivial case can be considered when the system is hydrologically closed (i.e. $P = 0$ and $T_r = 0$). In such a closed system, there is no hysteretic interaction between plant-water storage and soil moisture because $\frac{dW}{dV} = -\frac{a}{b}$. Thus, W must linearly relate to V. This can be written as:

$$V = -\frac{a}{b}W + k. \quad \text{(Eq. S3)}$$

This finding is compatible with the fact that in a ‘closed system’, the total water A_T must be conserved, meaning $W + V$ is a constant $(= A_T)$ at all times. However, this finding is opposite to the quasi-linear relation between W and V with a *positive slope* routinely reported in measurements and model results (Figure 6). This finding does not invalidate the predator-prey analogy but points to the obvious need for an open system with water loses.

Relaxing the closed system assumption by introducing transpirational losses in Eq. S2 is first considered. The focus is on a single dry-down event where $W(t)$ and $V(t)$ are both maximum after an extended rainfall event so that the maximum capacity of the stored water in the soil plant system is $A_T(0) = W(0) + V(0)$. Unlike the closed system case, $dA_T/dt = -T_r$ instead of zero. To proceed further, transpiration is assumed to be proportional to V and $\frac{a}{b} = 1$ must be invoked to satisfy conservation of mass. The predator-prey system becomes

$$\frac{dW}{dt} = -a\,WV \quad \text{(Eq. S4)}$$

$$\frac{dV}{dt} = +a\,WV - c\,V = aV\left(W - \frac{c}{a}\right), \quad \text{(Eq. S5)}$$

where c is a constant reflecting the transpirational losses. Dividing eqn. S5 by S4 to eliminate time, the autonomous dynamical system yields

$$\frac{dV}{dW} = -1 + \left(\frac{c}{a}\right)\frac{1}{W}. \qquad \text{(Eq. S6)}$$

Integrating this ordinary differential equation yields

$$V(t) - V(0) = W(0) - W(t) + \left(\frac{c}{a}\right) log\left[\frac{W(t)}{W(0)}\right], \qquad \text{(Eq. S3)}$$

where $W(0)$ and $\mathrm{V}(0)$ are the soil and plant water storage at the beginning of a dry-down. The hysteresis becomes evident because at a given time t, there are multiple values of $\mathrm{W}(t)$ that satisfy a single $\mathrm{V}(t)$. The so-called 'symmetric dynamics' between W and V in a closed system (eqn. s5) also suggests that losses must play a role in both the sign and deviations from linearity in the two-water pool system as evidenced by the role of $\left(\frac{c}{a}\right)$ in Eq. S6.

Pushing this analogy further, if $\left(\frac{c}{a}\right)\frac{1}{W} > 1$, then $\frac{dV}{dW} > 0$, suggestive of a positive slope and non-linear relation between W and V. However, when $\left(\frac{c}{a}\right)\frac{1}{W} < 1$, a decreasing W leads to increasing V. Such a case is expected when c takes on a zero value (at night with transpiration small) but a positive value during the day (higher transpiration). Obvious extensions are that c varies with time (due to fast drivers of transpiration such as vapor pressure deficit and photosynthetically active radiation), and transpiration is non-linearly related to S. These extensions do not alter the qualitative character of the analogy here to explain the hysteretic behavior.

As a bridge to Figure 6, Eq S6 is illustrated for an effective rooting zone depth of 0.5 m, soil porosity of 0.5, $W(0)$ set to 50% of the soil porosity, and $V(0)$ =10 kg m^{-2} with $\frac{c}{a} = 60$ kg

m^{-2} (Fig S1). The hysteresis emerges when noting that for a given $V(t)$, two possible $W(t)$ satisfy Eq. s6.

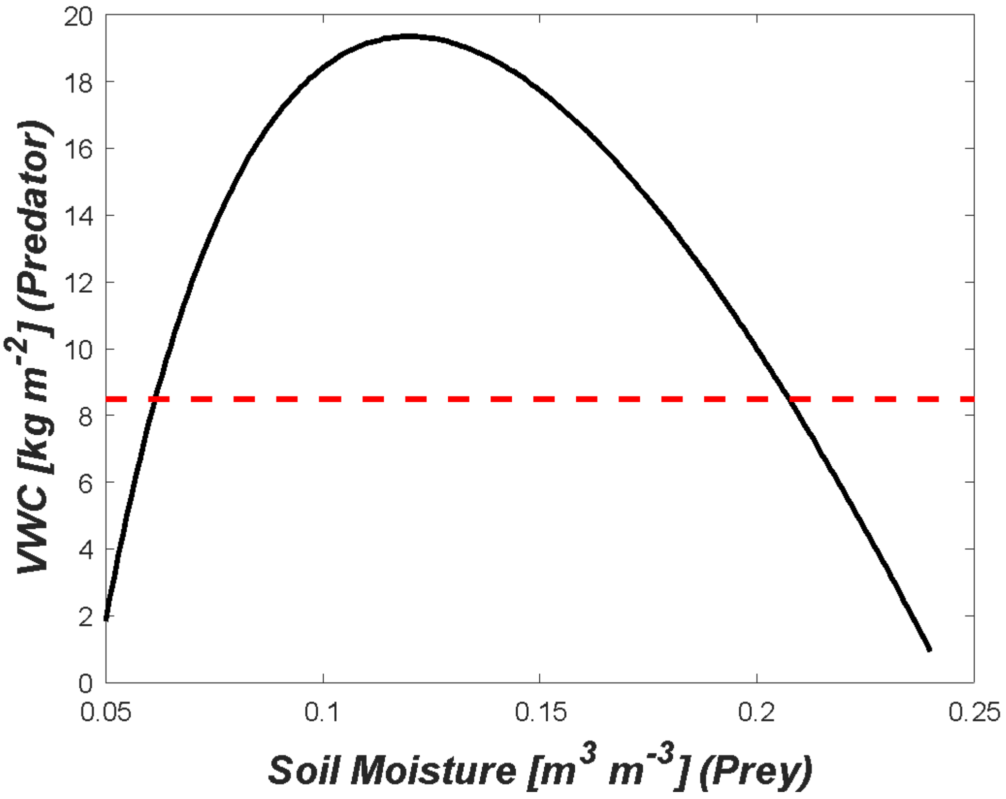

Fig. S1: Illustration of the theoretical relationship between VWC and soil moisture in the predator-prey analogy. For a given VWC value, there are multiple associated possible soil moisture values, giving rise to hysteresis. Multiple versions of this curve are possible depending on the initial conditions, but hysteresis is a constant feature.

<u>References</u>

Mrad, A., Katul, G. G., Levia, D. F., Guswa, A. J., Boyer, E. W., Bruen, M., et al. (2020). Peak grain forecasts for the US High Plains amid withering waters. *Proceedings of the National Academy of Sciences*, *117*(42), 26145–26150. https://doi.org/10.1073/pnas.2008383117

Wangersky, P. J. (1978). Lotka-Volterra population models. *Annual Review of Ecology and Systematics*, *9*(1), 189–218.
